# Training with noninvasive brain–machine interface, tactile feedback, and locomotion to enhance neurological recovery in individuals with complete paraplegia: a randomized pilot study

**DOI:** 10.1038/s41598-022-24864-5

**Published:** 2022-11-29

**Authors:** Miguel A. L. Nicolelis, Eduardo J. L. Alho, Ana R. C. Donati, Seidi Yonamine, Maria A. Aratanha, Guillaume Bao, Debora S. F. Campos, Sabrina Almeida, Dora Fischer, Solaiman Shokur

**Affiliations:** 1 Neurorehabilitation Laboratory, Associação Alberto Santos Dumont para Apoio à Pesquisa (AASDAP), São Paulo, 05440-000 Brazil; 2 Department of Neurobiology, Duke University Medical Center, Durham, NC 27710 USA; 3 Edmond and Lily Safra International Institute of Neuroscience of Natal, Macaíba, RN 59280-000 Brazil; 4 Clinics for Pain and Functional Neurosurgery, São Paulo, 01239-040 Brazil; 5 Associação de Assistência à Criança Deficiente (AACD), São Paulo, 05440-000 Brazil; 6 Hospital Israelita Albert Einstein, São Paulo, 05652900 Brazil; 7 Bertarelli Foundation Chair in Translational Neuroengineering, Neuro-X Institute, School of Engineering, Ecole Polytechnique Fédérale de Lausanne (EPFL), Lausanne, Switzerland; 8 Institute of BioRobotics and Department of Excellence in Robotics and AI, Scuola Superiore Sant’Anna, Pisa, Italy

**Keywords:** Regeneration and repair in the nervous system, Sensorimotor processing, Synaptic plasticity

## Abstract

In recent years, our group and others have reported multiple cases of consistent neurological recovery in people with spinal cord injury (SCI) following a protocol that integrates locomotion training with brain machine interfaces (BMI). The primary objective of this pilot study was to compare the neurological outcomes (motor, tactile, nociception, proprioception, and vibration) in both an intensive assisted locomotion training (LOC) and a neurorehabilitation protocol integrating assisted locomotion with a noninvasive brain–machine interface (L + BMI), virtual reality, and tactile feedback. We also investigated whether individuals with chronic-complete SCI could learn to perform leg motor imagery. We ran a parallel two-arm randomized pilot study; the experiments took place in São Paulo, Brazil. Eight adults sensorimotor-complete (AIS A) (all male) with chronic (> 6 months) traumatic spinal SCI participated in the protocol that was organized in two blocks of 14 weeks of training and an 8-week follow-up. The participants were allocated to either the LOC group (n = 4) or L + BMI group (n = 4) using block randomization (blinded outcome assessment). We show three important results: (**i**) locomotion training alone can induce some level of neurological recovery in sensorimotor-complete SCI, and (**ii**) the recovery rate is enhanced when such locomotion training is associated with BMI and tactile feedback (*∆*Mean Lower Extremity Motor score improvement for LOC =  + 2.5, L + B =  + 3.5; *∆*Pinprick score: LOC =  + 3.75, L + B =  + 4.75 and *∆*Tactile score LOC =  + 4.75, L + B =  + 9.5). (**iii**) Furthermore, we report that the BMI classifier accuracy was significantly above the chance level for all participants in L + B group. Our study shows potential for sensory and motor improvement in individuals with chronic complete SCI following a protocol with BMIs and locomotion therapy. We report no dropouts nor adverse events in both subgroups participating in the study, opening the possibility for a more definitive clinical trial with a larger cohort of people with SCI.

*Trial registration*: http://www.ensaiosclinicos.gov.br/ identifier RBR-2pb8gq.

## Introduction

Roughly 180,000 new spinal cord injuries (SCI) occur worldwide each year^1^. SCI causes a wide array of devastating motor, sensory, and autonomic deficits^2^. Additionally, SCI increases a person’s risk of developing secondary medical complications, ranging from hypotension and deep vein thrombosis to urinary tract infections, respiratory complications, and skin pressure ulcers^2^.

While the recovery of motor functions in individuals exhibiting the most severe cases of SCI (motor complete, American Spinal Injury Association (ASIA) Impairment Scale (AIS) A, AIS B^3^) is known to plateau within 1 year post-lesion, new emergent neurorehabilitation technologies have opened the potential of restoring motor^4–8^, sensory^9–12^, and visceral functions^13,14^ at least partially. Rehabilitation strategies can be divided into two approaches: (1) an *assistive* approach that helps the subject to regain a certain function (e.g., walking) through compensatory means, whereas (2) a *recovery* approach aims at achieving the restoration of the neurological functions that have been lost and/or damaged by the original spinal cord trauma or disease.

Examples of the assistive approach include most brain–machine interfaces (BMI) protocols for people with SCI. The strategy of such BMI-based protocols is to bypass the spinal cord lesion by extracting the individual’s brain signals related to motor intentions via either invasive (intracranially implanted electrodes^15^) or noninvasive (most often through electroencephalography (EEG)^16^) techniques (see^17^ for a review) and then using these neural signals to recover specific motor functions via, for example, direct control of a robotic orthosis^18,19^, an external robotic device^4,5^ or by the stimulation of the muscles (e.g., functional electrical stimulation, FES) below the level of the SCI lesion^20,21^.

During the past 5 years, we have reported multiple cases of significant neurological recovery in motor-complete SCI individuals using a multi-step noninvasive BMI-based protocol. These individuals followed a protocol integrating EEG-based brain–machine interfaces, visuo-tactile feedback (through the employment of immersive virtual reality environments) and assisted locomotion with multiple robotic gait training devices. After a 28-month training period with this protocol (known as the Walk Again Neurorehabilitation protocol or WANR), all participants (6 AIS A and 1 AIS B) recovered enough sensory-motor functions to be classified as AIS C^13^. The integration of the same noninvasive BMI and surface functional electrical stimulation (sFES) in a 5-month long protocol also showed a marked motor recovery below the lesion in one of the two participants who followed the training^22^. The hypothesis for such partial recovery comes from the observation that most individuals diagnosed as motor complete (AIS A) still have some intact nerve fibers of long spinal tracts, and consequently a subset of spinal cord connectivity, preserved at the level of the lesion (a condition referred to as *discomplete* lesion^23,24^).

Based on these previous studies, we raised the hypothesis that by engaging the SCI subject in a training protocol that aims at inducing cortical and spinal plasticity, it may be possible to target their spared spinal cord fibers and trigger the recovery of sensorimotor and visceral functions below the original spinal lesion. Interestingly, this hypothesis has also been supported by recent experimental studies in monkey and rat SCI models^25,26^. For example, studies with motor complete SCI rats showed that the integration of BMI and epidural electrical stimulation (EES), delivered through chronically implanted electrodes in the lumbar-sacral areas, enhanced neurological recovery compared with EES training alone^26^.

Despite these encouraging clinical observations, it remains unclear which components of the WANR protocol were fundamental for the induction of such a partial neurological recovery. In particular, it is still unknown whether the application of a protocol including only locomotion training, but not BMI and tactile feedback, could by itself explain the partial neurological recovery observed in our previous studies. Here we addressed this fundamental question by enrolling a second cohort of eight participants (all AIS A, 1–9 years post-lesion), randomly assigned to two groups; one in which four participants followed a training protocol involving only assisted locomotion (LOC group), and a second group in which participants performed the same locomotion training, along with BMI training that included the use of visuo-tactile feedback delivered by immersion in a virtual reality environment (L + B group). Our results demonstrated that while participants in both groups exhibited sensory and motor improvements below the level of the SCI, those belonging to the L + B group displayed higher levels of neurological recovery than the ones assigned to the LOC group.

## Materials and methods

### Inclusion criteria

Inclusion criteria for the study was adults (> 18 years old) with presence of chronic, traumatic lesion 6 months to 3 years prior to the onset of the protocol and ASIA impairment scales (AIS) A or B. We excluded individuals who had seizures; contractures in the legs (shortening of a muscle or tendon); cardiopulmonary instability; abuse of alcohol or drugs; psychiatric illness; history of head trauma with loss of consciousness within 3 months of recruitment; use of drugs known to exert negative effects on motor recovery; cognitive deficit; pregnancy or of childbearing potential and not using adequate contraception; presence of ferromagnetic material in the skull (except in the mouth); presence of cardiac and/or neural pacemakers; untreated depression; spasticity at the lower end of a Modified Ashworth Scale (MAS) score equal or higher than 2; uncontrolled diabetes; a degree of osteoporosis (T-score) greater than − 4.0; the presence of joint deformities, fractures, peripheral neuropathy of the upper limbs, brain injury, or amputation of upper or lower limbs; and those that did not have time for project activities or participated in another research project.

A total of 20 subjects were interviewed between February and June 2016; 19 from the medical files at the Associação de Assistência à Criança Deficiente, São Paulo, Brazil (AACD), and one from the Hospital Israelita Albert Einstein in São Paulo (see flow diagram in Fig. S1). The first evaluation was done by the clinical at the Associação Alberto Santos Dumont para Apoio à Pesquisa (AASDAP) over the phone. Six individuals were excluded as they did not have time to join the research or were living far from São Paulo. One individual had recurrent urinary tract infections due to long-term catheterization (catheter-associated urinary tract infection) and one had a skin lesion (pressure ulcers). The remaining 12 people were invited to undergo a standardized exam of sensorimotor functions in the lab [International Standards for Neurological Classification of SCI (ISNCSCI)] and a Magnetic Resonance Imaging (MRI) scan of the spinal cord. Among them, one was found to be emotionally unstable, one dropped out because of lack of time and two were found with severe syringomyelia—for these two individuals, we decided to avoid the risk of increasing the injury (especially during the body weight support training), given the proximity of the syringomyelia with the cervical spine. We enrolled the other eight subjects. The participants were all males classified as AIS A^3^ with traumatic SCI at the thoracic level (detailed Supplementary Table S1). They had no comorbidities and were all emotionally stable.

### Protocol design and randomization

The protocol design was a parallel two-arm randomized pilot study with two groups. The sample size was chosen based on previous results presented in Donati et al.^8^, where four out of eight motor-complete participants with SCI that trained for 12 months experienced significant neurological recovery. We used block randomization, with a sequentially numbered container generated with MATLAB. The participant enrollment, the random allocation, and participant assignment were done by the clinical responsible for the project. The outcome assessors were blinded to the protocol conditions.

### Study approval

The study was approved by both the local ethics committee (AACD, Brazil #364.027) and the Brazilian federal government ethics committee (CONEP, CAAE: 13165913.1.0000.0085). The protocol was part of a trial on the *Development of a Brain-controlled Gait Apparatus to Restore the Walking of People with Spinal Cord Injury* registered on the Brazilian Registry of Clinical Trials (http://www.ensaiosclinicos.gov.br/) with registration number RBR-2pb8gq on 18/09/2019 (registered retrospectively). All research activities were carried out in accordance with the guidelines and regulations of the AACD and CONEP. The participants signed a written informed consent before enrolling in the study and also signed written informed consent for open access publication (print and digital) of their images. The experiments were carried out at the AASDAP.

### Training protocol

**Figure 1:**
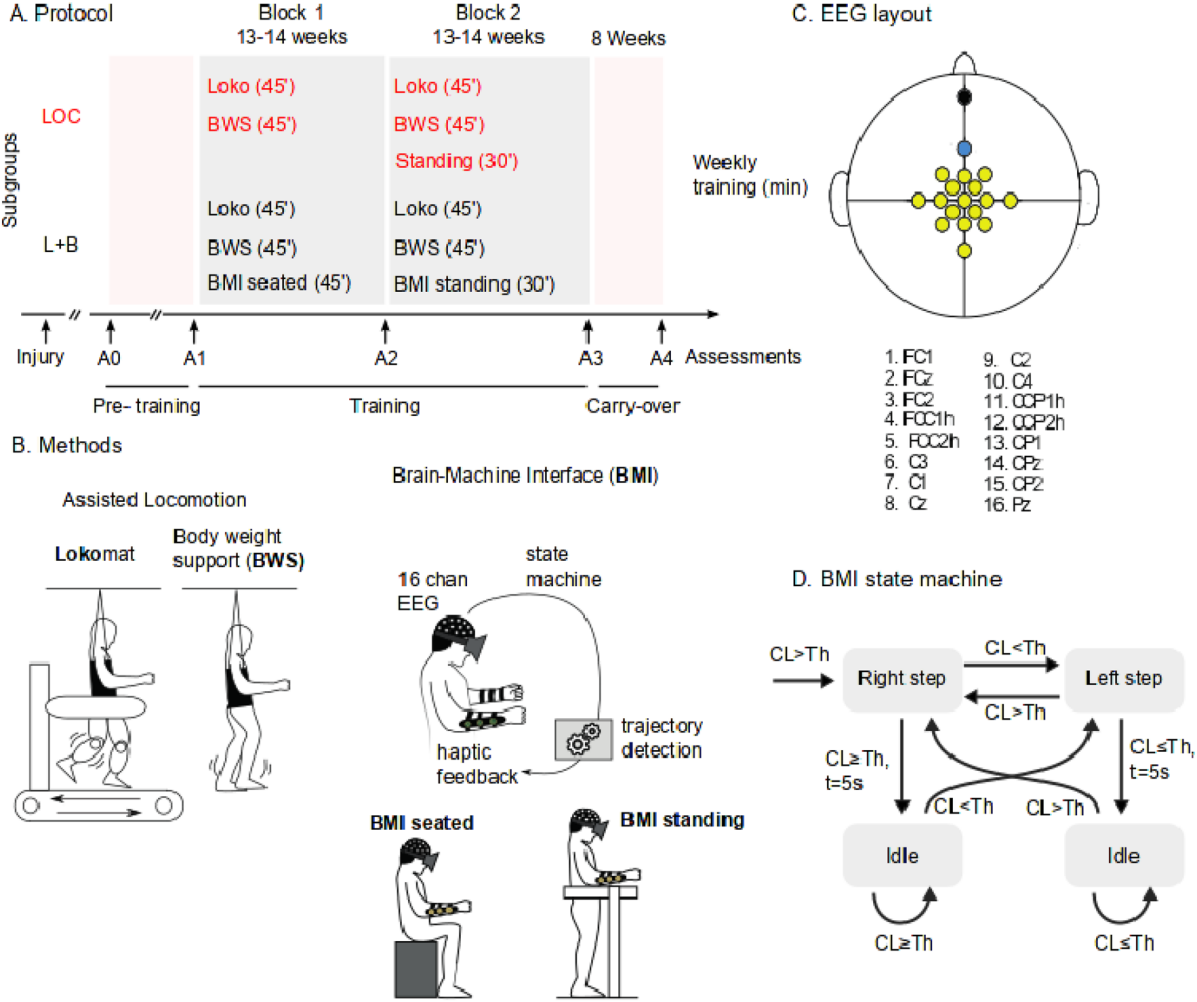
Experimental protocol. (**A**) Training protocol for the Locomotion only (red) and Locomotion + BMI (black) groups. A0 to A4 are clinical assessments. The Pre-training is the period going between the baseline measurement performed by the clinical institution that followed the individual before our protocol began and the onset of our protocol. The training phase contains two blocks of 13–14 weeks of training, and the carry-over phase goes between the end of the protocol and 8 weeks after the training. (**B**) Both LOC and L + B groups followed the assisted locomotion training, consisting of one Lokomat and one ZeroG training per week. The BMI training consisted of leg motor imagery to control an avatar and sensory feedback provided on subjects’ forearms (**C**) The 16 EEG electrode placement with the (R)eference and (G)round. (**D**) Left and right step animation on the avatar were triggered if the output of the linear Classifier (CL) was respectively below or above a certain threshold (− 1, + 1). If the subject did not alternate for 5 s, the avatar moved to an idle state.

Participants were randomly assigned to either a Locomotion only (LOC) or Locomotion + BMI (L + B) protocol. Both protocols consisted of two blocks of 13–14 weeks of training, with two training sessions per week (Fig. 1A). Both LOC and L + B groups followed the same assisted locomotion training (Fig. 1B) that we have previously described in detail^13^. In summary, it consisted of a 45-min workout per week with the Lokomat (Hocoma, AG, Volketswil), and a 45-min training session with a robotic bodyweight support system (ZeroG, Aretech LLC). Participant P3 had less ZeroG training due to ligament instability of the ankle; the locomotion training for this participant included a higher number of Lokomat training sessions. Overall, all participants had between 53 and 58 sessions of physical training considering the entire protocol (details in Supplementary Table S2). The L + B group did one session of BMI per week (Fig. 1B). For the BMI tasks during the first block (B1), they remained seated; during the second block (B2), they performed the BMI in a standing position (using an orthostatic table). The BMI and the Lokomat sessions were always done on the same day; the Lokomat training started 5–10 min after the end of the BMI session. To equalize the amount of physical training for both subgroups, LOC participants also had a 30-min weekly standing position session during B2: this session involved no BMI and no tactile feedback.

### Assessments

We performed four complete ISNCSCI assessments^3^ in the following order: (A1) onset of the training, (A2) end of Block 1, (A3) end of Block 2, and (A4) follow-up after an 8-week break. The A0 corresponded to the baseline measurement performed by the clinical institution that followed the individual within 1–6 years before our protocol began (see Supplementary Table S1). We also evaluated proprioception and perception of vibration (see^13^ for the description of the assessment) and the spinal cord MRI scans, using 1.5 T GE-Genesis equipment, with gadolinium-based intravenous contrast, at A1. Images were obtained in axial, sagittal and coronal planes and in T1, T2 and FIESTA (Fast Imaging Employing Steady-state Acquisition) sequences. The level of the anatomical lesions as revealed by the MRI is reported in Supplementary Table S1. Sensory evoked potential examination at A1 (Supplementary Table S3A, S3B) showed complete dysfunction of the somatosensory pathway below the lesion level; none of the participants had evoked somatosensory potentials in the lower limbs.

### EEG recording and BMI task

The EEGs were collected using a noninvasive EEG head cap worn by participants during each session (Brain Products GmbH. actiCAP). We employed 16-channel EEG recordings clustered around the midline, placed over the putative leg motor representation area (Fig. 1C). Raw scalp EEGs were collected using the V-Amp amplifier (BrainProduct) recording system and interfaced via USB to a computer. OpenViBE 0.16.0^27^, an open-source BMI software, was used for signal acquisition and filtering.

Each session started with an 8-min *calibration*. This routine started with a 30-s blank screen, during which participants were encouraged to relax. Soon after, either a left or a right arrow appeared on the screen. Participants were instructed to imagine making leg movements to the side corresponding to the direction pointed by the arrow on the screen. Subjects were encouraged to imagine single-leg movements related to kicking a soccer ball or movements associated with rotating their ankles; they were instructed not to think about cyclic bipedal movement. Each trial was followed by a 1–3 s intertrial period during which participants could readjust themselves. Forty trials (20 left, 20 right) were conducted per classifier training, lasting approximately 8 min in total. The raw signals were sampled at 2000 Hz and processed by applying a zero-phase band-pass Butterworth filter from 8 to 30 Hz, capturing alpha and beta waves. We measured the power spectrum of the epoched multidimensional time series (1-s window length with a 0.0625-s interval between consecutive windows) and used the Common Spatial Pattern (CSP) for feature extraction. Finally, we used a linear discriminant analysis (LDA) over the 6-dimensional features extracted by the CSP to construct the EEG classifier. Each classifier was scored based on fivefold cross-validation. A second training session was run if the classifier score was lower than 70%.

The *BMI training* consisted of alternating left and right leg motor imagery (MI) to trigger the corresponding stepping of a 3D avatar projected in a head-mounted display (Oculus Rift DK1) showing a virtual reality environment^1^ (Supplementary Fig. S2A). Each session consisted of four blocks of 6 min. We used the CSP and LDA parameters calculated during the calibration phase to decode the MI and control the stepping of the avatar. A custom C++ code running at 100 Hz updated the avatar movements (Fig. 1D) based on the classifier output with a moving average over 500 ms. The avatar steps were chosen to be small to ensure good visibility of the legs during the walk: the steps were 7.25 cm long. If the participant stopped alternating for at least 5 s, a walk-to-stop animation was triggered, and the avatar moved to an idle position. After the idle state, the participant had to trigger the step contralateral to the step before the stop: this prevented that non-alternating strategy to result in a step–stop–step–stop loop. The participant was instructed to perform as many steps as possible and to stop when randomly placed stop cues appeared in the virtual environment. Stop cues were 30 cm wide and randomly spaced by 80–130 cm from each other. Example of one trial is shown on supplementary Movie S1. We measured the BMI performance considering both the distance walked by the participants and their ability to stop when instructed (Supplementary Fig. S2B for details of the performances). The subjects’ performance was compared to a random walk (see Supplementary Fig. S3 for detail of measurement of chance level).

*Tactile feedback* was provided via a portable haptic device that we have described in detail elsewhere^2,3^; it consists of arrays of small vibrating elements aligned on the participants’ forearm skin surface on top of their ulna. Tactile feedback informed subjects of the virtual legs’ swing state (apparent movement from elbow to the wrist lasting 2000 ms) and the onset of the gait stance event (short 600 ms buzzing on the ipsilateral arm).

## Results

### BMI results

**Figure 2:**
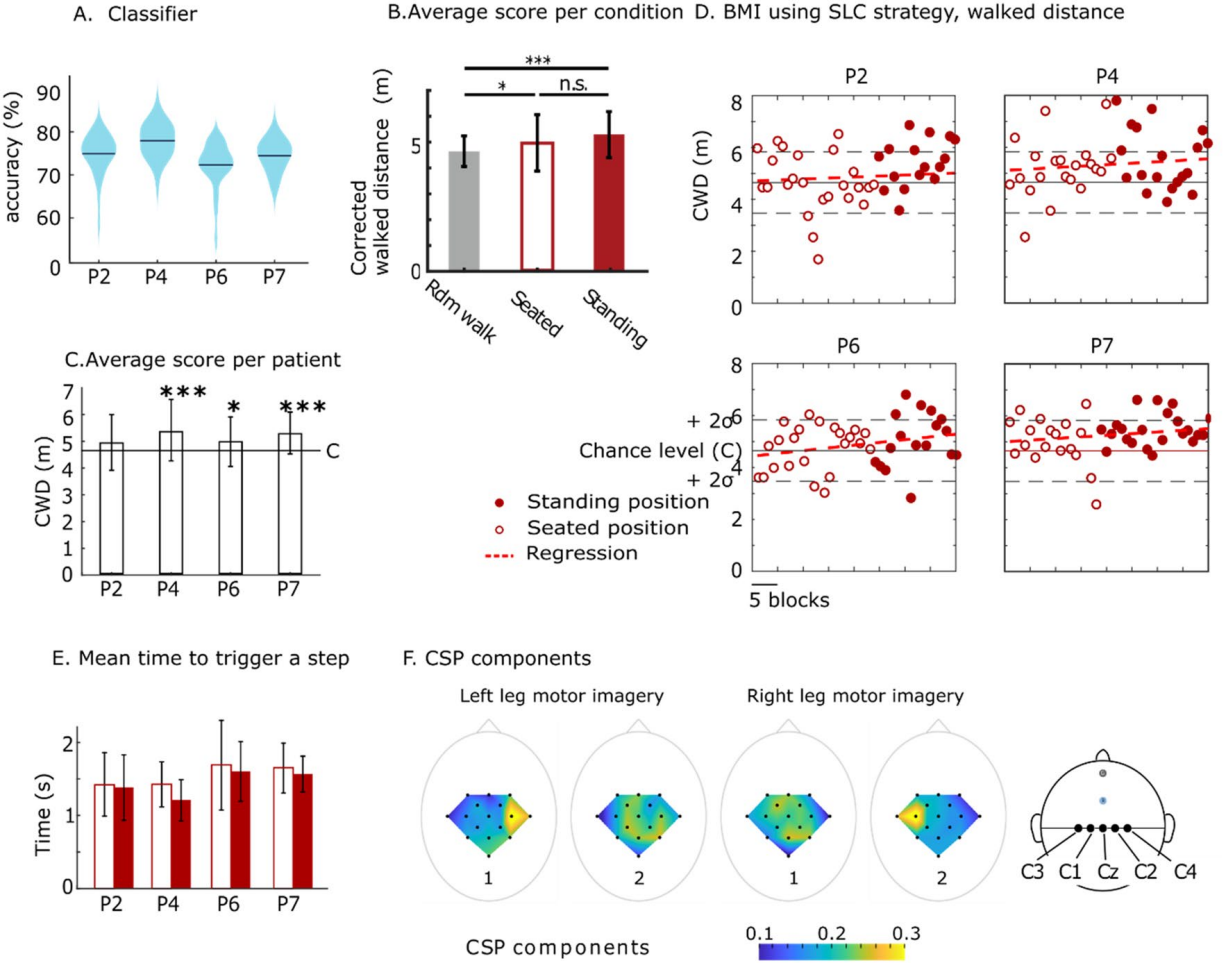
BMI scores. (**A**) EEG classifier accuracy for the calibration sessions. (**B**) The mean corrected walk distance (CWD) for BMI sessions performed when the subject was seated (empty red bar) or standing position (red filled bar). Both conditions are compared to random (see Supplementary Fig. S1 for description). *P < 0.05, **P < 0.01. Bars are stand deviation (SD) (**C**) Mean and SD for the CWD for the four study participants that followed the BMI protocol considering both seated and standing sessions and compared to chance level (**D**) CWD for each block of 6 min of BMI performed seated (empty red circles) or standing (filled red circles), compared to chance level (black line) as measured by the random walk. *P < 0.05, **P < 0.01, *P* < 0.001, t-test. (**E**) Mean ± SD time to trigger a step during BMI tasks. (**F**) The Average coefficients for the first and second CSP component considering the training sessions for the four participants that followed the BMI protocol.

Figure 2A–D describe the results obtained during the BMI interaction. The classifier accuracy—obtained during the calibration phase—was on average above 72% for all participants (Fig. 2A, P2 = 75.58%, P4 = 78.63%, P6 = 72.89%, P7 = 75.11%), which is above the classification rate to assert statistical significance (= 62.5%, binomial cumulative distribution function for 40 trials, *P* value < 0.05^4^).

We report the corrected walk distance—calculated as the walked distance during a block, minus the sum of missed stop cues x the cues size (Fig. S2B)—per 6-min training blocks. A one-way ANOVA was conducted to compare the avatar walking performance in seated and standing positions to a random walk. We found a significant effect on avatar walking performance for the three conditions (F(2,279) = 10.08, *P* = 0). Post hoc comparisons using Bonferroni correction indicated that the mean score for both ‘standing’ and ‘seated’ conditions was significantly higher than chance level (Fig. 2B). Furthermore, we detected no difference between the ‘standing’ and ‘seated’ conditions, indicating that participants controlled the walk of the avatar legs equally well in both situations.

Performances for P4, P6, and P7 for BMI control were found to be significantly above chance level (t-test, *P* < 0.05) (Fig. 2C). Steady performance improvements were observed throughout the session for P4 and P6 (Fig. 2D), and P7. Participant P2 was the only one to stagnate over time and who did not reach significance (t-test, *P* = 0.07). This individual was found to be, on average, quicker than P6 and P7 to trigger a step, with an average time to perform the task under 1.5 [s] (Fig. 2E). Participant P4’s average time to initiate the step was also under 1.5 [s], making him both a fast and good performer. The times for P6 and P7 were under 2 [s] on average.

Figure 2F shows the CSP coefficients^28^, averaged over all sessions, for all participants recorded during the classifier training phase. Overall, the coefficients of the CSP filter permitted identifying the contribution of each individual EEG electrode to the classification. The highest contribution for left leg motor imagery was found on the C2 electrode and on the C1 for the right side. These results confirmed that the larger contribution was obtained from the electrodes close to the midline and suggests that subjects were using leg motor imagery to operate the BMI apparatus.

### Clinical improvement

**Figure 3:**
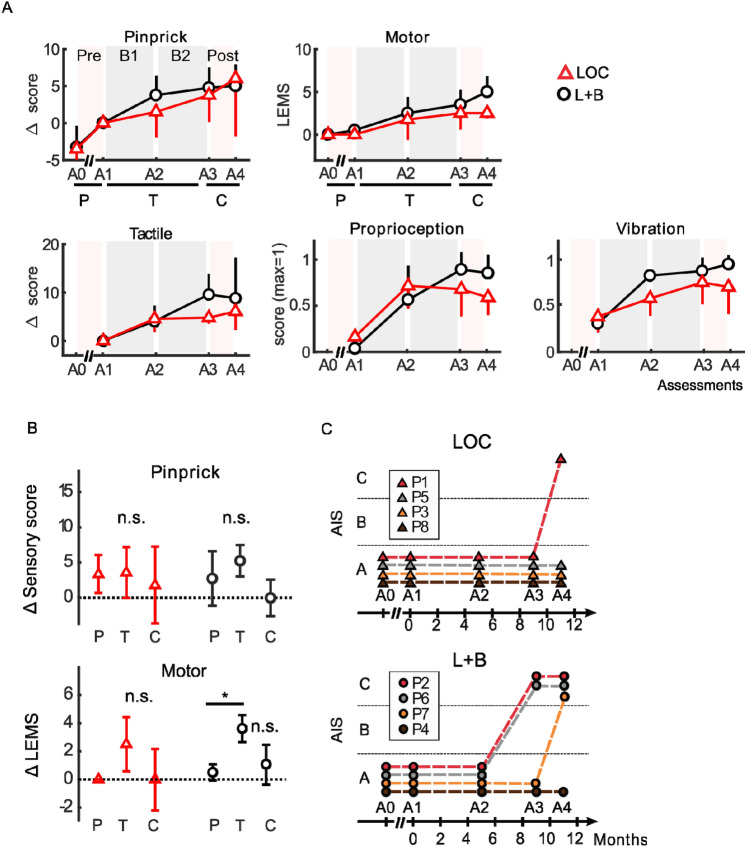
Clinical outcome. (**A**) Mean ± STD clinical scores (n = 4) for the subjects following the locomotion + BMI training (black circle) and the locomotion only protocol (red triangles). Pinprick's score and tactile score are reported as differences in the baseline assessment at the onset of the protocol (A1); for motor performance, we show the raw lower extremity motor score (LEMS^3^). We also report the lower limb proprioception score and vibration score (max score = 1). The measurement A0 was done (by a different clinical institution) 1–3 years before the onset of the protocol and consisted of only pinprick and motor measurements. (**B**) Improvement (sensory or LEMS score) measured for participants following the L + B (black circles) or LOC (red triangle) groups for (P)re training (improvement between A0 and A1), (T)raining (improvement between A1 and A3) and (C)arry over (between A3 and A4) (Friedman test, and multiple comparisons with Bonferroni correction, *P < 0.05). (**C**) AIS grades for all study participants.

Tables 1 and 2 disclose the clinical scores for sensory and motor modalities for participants in both groups. We found steady improvement throughout the protocol (A1–A3) in both the motor and sensory domains for the two groups (Fig. 3A). We also observed a higher delta score in A3 for the L + B group compared to the LOC group for the Pinprick test (L + B = 4.75, LOC = 3.75), tactile (L + B = 9.5, LOC = 4.75), LEMS (L + B = 3.5, LOC = 2.5), proprioception (L + B = 0.88, LOC = 0.66) and vibration (L + B = 0.75, LOC = 0.70).

**Table 1:** Clinical scores for the LOC subgroup.

	Tactile (LT)				Nociception (PP)				Motor (LEMS)			
	A1	A2	A3	A4	A1	A2	A3	A4	A1	A2	A3	A4
P1	58	61	63	63	58	58	65	67	0	2	4	2
P3	50	55	55	56	51	49	54	50	0	0	0	3
P5	44	52	50	55	44	50	50	59	0	0	2	2
P8	68	70	71	70	69	71	68	69	0	5	4	3
Mean	55	59.5	59.75	61	55.5	57	59.25	61.3	0	1.75	2.5	2.5

**Table 2:** Clinical scores for the L + B subgroup.

	Tactile (LT)				Nociception (PP)				Motor (LEMS)			
	A1	A2	A3	A4	A1	A2	A3	A4	A1	A2	A3	A4
P2	64	68	76	73	64	68	72	69	1	4	5	4
P4	64	70	71	68	65	70	68	67	1	4	5	7
P6	75	75	80	78	75	75	77	79	0	2	4	6
P7	61	69	75	82	61	67	67	70	0	0	2	3
Mean	66	70.5	75.5	75.25	66.25	70	71	71.3	0.5	2.5	4	5

Next, we compared the improvement rates during (T)raining (A3–A1 in Fig. 3B) to the spontaneous recovery registered in the (P)re-training phase (A1–A0), and the (C)arry-over effect after the protocol (A4–A3). In the sensory domain, no group differences were found for the LOC (Friedman test, *P* = 0.82) and the L + B (*P* = 0.11) groups. In the motor domain, we found a significant difference for the L + B group (Friedman test, *P* = 0.04) and none for the LOC group (*P* = 0.26). Further analysis showed a significant difference between T and P conditions (*P* < 0.05, multiple comparisons with Bonferroni correction), but not between T and C (*P* > 0.1).

By the end of the protocol, 3 out of 4 L + B participants had recovered enough functions to be classified as AIS C (Fig. 3C, Tables S4–S11, Supplementary Movie S2). On the other hand, one participant from the LOC group converted from AIS A to AIS C. While clearly encouraging, these results should be taken with precaution given the small sample size and differences between the two subgroups. Indeed, the L + B group had a higher clinical score at the onset of the training (A1) (Tables 1 and 2) compared to the LOC group which could, in part, explain the difference observed in the AIS scores.

## Discussion

We carried out a randomized pilot study to investigate the effect of assisted locomotion training plus noninvasive BMI and tactile feedback to induce partial neurological recovery for people with the most severe loss of sensory and motor functions (i.e., AIS A) due to spinal cord injury. We observed improvement rates in motor functions incompatible with spontaneous recovery. To our knowledge, the present study is the first to perform a randomized pilot study that investigates locomotion training in combination with noninvasive BMI for the rehabilitation of individuals with motor-complete SCI. Overall, we report two important results; first, sustained assisted locomotion training induces partial sensory and motor recovery even at the chronic phase of the lesion (in our case, between 1 and 8 years after the spinal cord injury). This finding is coherent with the results by Van den Brand and colleagues^29^ that showed that training with the subject's active participation induced extensive remodeling of cortical projections to subcortical targets, including brainstem structures and intraspinal relay regions. Second, we observed that the BMI protocol amplified this partial neurological recovery effect. These results are coherent with recent studies with rats^5^ and non-human primate models^6^.

We also found that the participants were able to learn the BMI task consisting of motor imagery of single leg movement. Indeed, this cortical activation was confirmed by the modulation of the EEG signal obtained from the electrodes C1 and C2, which were placed over the putative leg representation areas (with lateralization) in the primary motor and somatosensory cortices.

### Possible neurological mechanism

But how can one explain our results? In our study, the BMI approach was used as a way to provide users with neurofeedback signals correlated to their production of the leg motor imagery. The subjects had to perform left and right leg motor imagery, primarily using their primary somatosensory and motor cortices, to control the movements of avatar legs in a virtual environment, while coherent visual and tactile feedback signals provided the closing of the control loop created by the BMI paradigm. We propose that this BMI training, which preceded the training with the robotic gait training, helped the participants to regain the ability to activate the leg motor areas of their sensorimotor cortex. As such, in our view, the motor recovery that has been reported in both groups, i.e., locomotion only and locomotion + BMI groups, likely emerged due to the reactivation of cortical circuits that remained dormant after the spinal cord injury. Partial cortical reactivation was likely possible just with active locomotion training with the robotic gait trainer and the bodyweight support. However, when this was combined with BMI training, cortical activation was likely stronger and more sustained. As such, participants’ improvements were larger in the group that performed the BMI task than the one in which only active locomotion was carried out.

This vision is justified by a series of well-established findings. Indeed, it is now well-documented that long-term training with BMIs promotes brain plasticity^15,30^. Such a conclusion is warranted by numerous clinical studies in people with stroke that have shown significant motor recovery following a protocol integrating EEG-based BMIs and physical training^31,32^. A recent study showed that EEG-based training increased functional connectivity between motor areas in the affected hemisphere^33^. Similarly, studies from our group^8,13,22^ and others^25,26,34^ have shown neurological recovery in SCI subjects following a long-term BMI-based training protocol.

### Limitations and future directions

One major limitation of the current study was the small number of participants. This pilot study is nevertheless essential to justify future studies with a larger cohort. In addition to the a standardized exam of sensorimotor functions [International Standards for Neurological Classification of SCI (ISNCSCI)], baseline measurement should include neurophysiological measures such as the brain motor control assessment (BMCA)^1^. A second limitation was the lack of blinding of the participants and caregivers. We cannot exclude the possible positive effects due to higher concentration or higher motivation in the BMI group; future protocols should therefore include a sham BMI group (as used in^31^).

Next, while important levels of sensory recovery were observed, the results did not reach statistical significance when compared to the period before the onset of the protocol. This was because some levels of spontaneous recovery were registered during the Pre-training period (a period going from several years before the onset of the protocol to the onset of the protocol). One possibility that we could not test is that this spontaneous improvement happened in the early phase of Pre-training and that by the time of the protocol, the participants had reached their plateau. In this hypothesis, our protocol might have triggered sensory recovery after a plateau was already reached.

While in the current protocol, participants’ improvements were not large enough to translate into improvement in walking, two aspects of the protocol could be adapted to promote neurological recovery and potentially any functional recovery. First, the training protocol could be more intense (for example, increasing the number of training sessions to 4 days per week). Next, our WANR protocol could be introduced early on after an SCI lesion (at the subacute phase of the lesion) and continuing until a plateau of functional and clinical recovery is reached. Indeed, during this period (2 and 12 months after an SCI occurrence) there is an intense period of neurological reorganization at the level of the spinal cord lesion^35^.

To conclude, we have investigated a protocol integrating noninvasive BMIs and locomotion therapy for people with chronic complete spinal cord injury. Our study showed that it was possible to improve sensory and motor functions in individuals with severe SCI lesions, even at the chronic phase of the lesion. Throughout the protocol, we reported no adverse events or dropouts, suggesting that the training was safe and likely motivating for the participants. Further clinical trials with larger cohorts should be done to confirm the potential of this protocol to induce neurological and possibly functional recovery in people with SCI.

## Supplementary Information

Supplementary Information 1.

Supplementary Information 2.

Supplementary Information 3.

Supplementary Movie S1.

Supplementary Movie S2.
