# Supplementary material for: Training with noninvasive brain–machine interface, tactile feedback, and locomotion to enhance neurological recovery in individuals with complete paraplegia: a randomized pilot study
Source: Sci Rep. 2022 Nov 29;12:20545. doi: 10.1038/s41598-022-24864-5 (PMC9709065; doi:10.1038/s41598-022-24864-5)
Supplement: Supplementary file 1 — Supplementary Information 1. [file 41598_2022_24864_MOESM1_ESM.docx]

**Training with noninvasive brain-machine interface, tactile feedback, and locomotion to enhance neurological recovery in individuals with complete paraplegia: a randomized pilot study**

Miguel A. L. Nicolelis^1-3*^, Eduardo J. L. Alho^1,4^, Ana R. C. Donati^1,5^, Seidi Yonamine^1,3^, Maria A. Aratanha ^1,6^, Guillaume Bao^1^, Debora S. F. Campos^1,6^, Sabrina Almeida^1,5^, Dora Fischer ^1,5^, Solaiman Shokur ^1,7,8^

^1^ Neurorehabilitation Laboratory, Associação Alberto Santos Dumont para Apoio à Pesquisa (AASDAP), São Paulo, Brazil, 05440-000;

^2^ Emeritus Professor, Department of Neurobiology, Duke University Medical Center, Durham, NC, 27710;

^3^ Edmond and Lily Safra International Institute of Neuroscience of Natal, Macaíba - RN, Brazil, 59280-000;

^4^ Clinics for Pain and Functional Neurosurgery, São Paulo, Brazil, 01239-040;

^5^ Associação de Assistência à Criança Deficiente (AACD), São Paulo, Brazil,05440-000;

^6^ Hospital Israelita Albert Einstein, São Paulo, Brazil, 05652900;

^7^ Bertarelli Foundation Chair in Translational Neuroengineering, Center for Neuroprosthetics and School of Engineering, Ecole Polytechnique Fédérale de Lausanne (EPFL), Lausanne, Switzerland

^8^ Institute of BioRobotics and Department of Excellence in Robotics and AI, Scuola Superiore Sant’Anna, Pisa, Italy

* Corresponding author: [nicoleli@neuro.duke.edu](mailto:nicoleli@neuro.duke.edu)

**Supplementary Tables**

**Table S1.** Participants’ demography. ^1^ASIA Impairment Scale ^2^Anatomic lesion level ^3^MRI guided. Time since lesion in years, except for participant P6, who had the lesion ten months before the protocol onset. ^5^ Etiology, C: Closed Trauma; O: open injury. ^6^ Time in months between the baseline measurement done by the clinical institution that followed the participants (A0) and the measurement done at the onset of our protocol (A1)

| - **Subject** | - **Protocol** | - **Sex** | - **Age** | - **AIS^1^** | - **Lesion Level** - **Clinic^2^ Right Left** | | - **Lesion Level MRI^3^** | - **Time since lesion^4^** | - **Etiol^5^** | - **Time A0 –A1^6^** |
| --- | --- | --- | --- | --- | --- | --- | --- | --- | --- | --- |
| - **P1** | - LOC | - M | - 23 | - A | - T5 | - T6 | - T5-T7 | - 6 | - O | - 47 |
| - **P2** | - L+B | - M | - 28 | - A | - T8 | - T8 | - T7-T10 | - 6 | - C | - 36 |
| - **P3** | - LOC | - M | - 34 | - A | - T5 | - T5 | - T4-T8 | - 3 | - C | - 15 |
| - **P4** | - L+B | - M | - 48 | - A | - T8 | - T8 | - T8-T11 | - 7 | - C | - 73 |
| - **P5** | - LOC | - M | - 30 | - A | - T3 | - T3 | - T2-T6 | - 6 | - C | - 37 |
| - **P6** | - L+B | - M | - 28 | - A | - T12 | - T12 | - T9-L1 | - 10 m | - O | - 6 |
| - **P7** | - L+B | - M | - 19 | - A | - T8 | - T6 | - T5-T7 | - 5 | - C | - 37 |
| - **P8** | - LOC | - M | - 31 | - A | - T8 | - T9 | - T8-T12 | - 8 | - O | - 75 |

**Table S2.** Number of sessions of training per participant for the WANR protocol. All participants had a similar amount of Locomotion and Standing training (orthostatism).

|  | Lokomat training | ZeroG training | Sum  locomotion training | Orthostatism | Sum BMI training |
| --- | --- | --- | --- | --- | --- |
| P1 | 28 | 28 | 56 | 25 | 0 |
| P2 | 27 | 30 | 57 | 24 | 24 |
| P3 | 33 | 22 | 55 | 25 | 0 |
| P4 | 30 | 26 | 56 | 26 | 26 |
| P5 | 29 | 29 | 58 | 23 | 0 |
| P6 | 28 | 25 | 53 | 26 | 26 |
| P7 | 26 | 28 | 54 | 25 | 25 |
| P8 | 27 | 26 | 53 | 25 | 0 |

Table S3. Somatosensory evoked potential evaluation done at A1 for all the participants (see Supplementary Table S3.xlsx).

**Supplementary Tables**

**Table S4.** ISNCSCI assessment for participant P1 done at the onset of training (A1), at the end of the first block of 16 weeks of training (A2), and the end of the second block of 16 weeks of training (A3), and after an 8-week break (A4). LT: Light Touch; PP: Pin Prick; DAP: Deep anal pressure; VAC: Voluntary anal contraction; UER/UEL: Upper Extremity Right/Left; LER/LEL: Lower Extremity Right/Left; UEMS/LEMS: Upper/Lower Extremity Motor Score; ZPP: Zone of Partial Preservation

|  |  | **A1** | | **A2** | | **A3** | | **A4** | |
| --- | --- | --- | --- | --- | --- | --- | --- | --- | --- |
|  |  | **Right** | **Left** | **Right** | **Left** | **Right** | **Left** | **Right** | **Left** |
| LT | S4-S5 | 0 | 0 | 0 | 0 | 0 | 0 | 1 | 1 |
|  | **RLT + LLT** | **29** | **29** | **30** | **31** | **31** | **32** | **31** | **32** |
|  | **LT TOTAL** | **58** | | **61** | | **63** | | **63** | |
| **PP Prick** | S4-S5 | 0 | 0 | 0 | 0 | 0 | 0 | 1 | 1 |
|  | **RPP + LPP** | **29** | **29** | **29** | **29** | **35** | **30** | **33** | **34** |
|  | **PP TOTAL** | **58** | | **58** | | **65** | | **67** | |
|  | **DAP** | No | | No | | No | | Yes | |
|  | **VAC** | No | | No | | No | | No | |
|  |  |  |  |  |  |  |  |  | |
| **Upper extr.** | **C5 - Elbow flexors** | 5 | 5 | 5 | 5 | 5 | 5 | 5 | 5 |
|  | **C6 - Wrist extensors** | 5 | 5 | 5 | 5 | 5 | 5 | 5 | 5 |
|  | **C7 - Elbow extensors** | 5 | 5 | 5 | 5 | 5 | 5 | 5 | 5 |
|  | **C8 - Finger flexors** | 5 | 5 | 5 | 5 | 5 | 5 | 5 | 5 |
|  | **T1 - Finger abductors** | 5 | 5 | 5 | 5 | 5 | 5 | 5 | 5 |
|  |  |  |  |  |  |  |  |  | |
| **Lower extr.** | **L2 - Hip flexors** | 0 | 0 | 0 | 0 | 1 | 1 | 1 | 1 |
|  | **L3 - Knee extensors** | 0 | 0 | 0 | 1 | 1 | 1 | 0 | 0 |
|  | **L4 - Ankle dorsiflexors** | 0 | 0 | 0 | 0 | 0 | 0 | 0 | 0 |
|  | **L5 - Long toe extensors** | 0 | 0 | 0 | 0 | 0 | 0 | 0 | 0 |
|  | **S1 - Ankle plantar flexors** | 0 | 0 | 1 | 0 | 0 | 0 | 0 | 0 |
|  |  |  |  |  |  |  |  |  | |
|  | **UER - UEL** | **25** | **25** | **25** | **25** | **25** | **25** | **25** | **25** |
|  | **UEMS TOTAL** | **50** | | **50** | | **50** | | **50** | |
|  | **LER - LEL** | **0** | **0** | **1** | **1** | **2** | **2** | **1** | **1** |
|  | **LEMS TOTAL** | **0** | | **2** | | **4** | | **2** | |
|  |  |  |  |  |  |  |  |  | |
| **Neuro. Levels** | **Sensory** | T7 | T7 | T7 | T7 | T7 | T7 | T7 | T7 |
|  | **Motor** | T7 | T7 | T7 | T7 | T7 | T7 | T7 | T7 |
|  | **Neurological Level of Injury** | T7 | | T7 | | T7 | | T7 | |
|  | **Complete or Incomplete** | C | | C | | C | | I | |
|  | **ASIA Impairment Scale** | A | | A | | A | | C | |
|  | **ZPP Sensory** | T8 | T8 | T9 | T10 | S3 | S3 | S5 | S5 |
|  | **ZPP Motor** | T7 | T7 | S1 | L3 | L3 | L3 | L2 | L2 |
|  | **Comments** |  | |  | |  | | Deep anal pressure present and motor function present in more than three segments (L2) below the motor level (T7) | |

**Table S5.** ISNCSCI assessment for participant P2 done at the onset of training (A1), at the end of the first block of 16 weeks of training (A2) and the end of the second block of 16 weeks of training (A3), and after an 8-week break (A4). LT: Light Touch; PP: Pin Prick; DAP: Deep anal pressure; VAC: Voluntary anal contraction; UER/UEL: Upper Extremity Right/Left; LER/LEL: Lower Extremity Right/Left; UEMS/LEMS: Upper/Lower Extremity Motor Score; ZPP: Zone of Partial Preservation.

|  |  | **A1** | | **A2** | | **A3** | | **A4** | | |
| --- | --- | --- | --- | --- | --- | --- | --- | --- | --- | --- |
|  |  | **Right** | **Left** | **Right** | **Left** | **Right** | **Left** | **Right** | | **Left** |
| LT | S4-S5 | 0 | 0 | 0 | 0 | 0 | 0 | 1 | | 1 |
|  | **RLT + LLT** | **32** | **32** | **34** | **34** | **38** | **38** | **37** | | **36** |
|  | **LT TOTAL** | **64** | | **68** | | **76** | | **73** | | |
| **PP Prick** | S4-S5 | 0 | 0 | 0 | 0 | 0 | 0 | 1 | | 1 |
|  | **RPP + LPP** | **32** | **32** | **34** | **34** | **36** | **36** | **35** | | **34** |
|  | **PP TOTAL** | **64** | | **68** | | **72** | | **69** | | |
|  | **DAP** | No | | No | | Yes | | Yes | | |
|  | **VAC** | No | | No | | No | | Yes | | |
|  |  |  |  |  |  |  |  |  | | |
| **Upper extr.** | **C5 - Elbow flexors** | 5 | 5 | 5 | 5 | 5 | 5 | 5 | 5 | |
|  | **C6 - Wrist extensors** | 5 | 5 | 5 | 5 | 5 | 5 | 5 | 5 | |
|  | **C7 - Elbow extensors** | 5 | 5 | 5 | 5 | 5 | 5 | 5 | 5 | |
|  | **C8 - Finger flexors** | 5 | 5 | 5 | 5 | 5 | 5 | 5 | 5 | |
|  | **T1 - Finger abductors** | 5 | 5 | 5 | 5 | 5 | 5 | 5 | 5 | |
|  |  |  |  |  |  |  |  |  |  | |
| **Lower extr.** | **L2 - Hip flexors** | 0 | 0 | 1 | 1 | 1 | 1 | 1 | 1 | |
|  | **L3 - Knee extensors** | 0 | 1 | 1 | 1 | 1 | 1 | 1 | 1 | |
|  | **L4 - Ankle dorsiflexors** | 0 | 0 | 0 | 0 | 0 | 0 | 0 | 0 | |
|  | **L5 - Long toe extensors** | 0 | 0 | 0 | 0 | 0 | 0 | 0 | 0 | |
|  | **S1 - Ankle plantar flexors** | 0 | 0 | 0 | 0 | 0 | 1 | 0 | 0 | |
|  |  |  |  |  |  |  |  |  | | |
|  | **UER - UEL** | **25** | **25** | **25** | **25** | **25** | **25** | **25** | **25** | |
|  | **UEMS TOTAL** | **50** | | **50** | | **50** | | **50** | | |
|  | **LER - LEL** | **0** | **1** | **2** | **2** | **2** | **3** | **2** | **2** | |
|  | **LEMS TOTAL** | **1** | | **4** | | **5** | | **4** | | |
|  |  |  |  |  |  |  |  |  | | |
| **Neuro. Levels** | **Sensory** | T8 | T8 | T8 | T8 | T8 | T8 | T8 | T8 | |
|  | **Motor** | T8 | T8 | T8 | T8 | T8 | T8 | T8 | T8 | |
|  | **Neurological Level of Injury** | T8 | | T8 | | T8 | | T8 | | |
|  | **Complete or Incomplete** | C | | C | | I | | I | | |
|  | **ASIA Impairment Scale** | A | | A | | C | | C | | |
|  | **ZPP Sensory** | T10 | T10 | T12 | T12 | S3 | S3 | S4-5 | S4-5 | |
|  | **ZPP Motor** | T8 | L3 | L3 | L3 | L3 | S1 | L3 | L3 | |
|  | **Comments** |  | |  | | Deep anal pressure present and motor function present more than three segments (S1) below motor level (T8) | | Deep anal pressure present and voluntary anal contraction. | | |

**Table S6.** ISNCSCI assessment for participant P3 done at the onset of training (A1), at the end of the first block of 16 weeks of training (A2), and the end of the second block of 16 weeks of training (A3), and after an 8-week break (A4). LT: Light Touch; PP: Pin Prick; DAP: Deep anal pressure; VAC: Voluntary anal contraction; UER/UEL: Upper Extremity Right/Left; LER/LEL: Lower Extremity Right/Left; UEMS/LEMS: Upper/Lower Extremity Motor Score; ZPP: Zone of Partial Preservation.

|  |  | **A1** | | **A2** | | **A3** | | **A4** | |
| --- | --- | --- | --- | --- | --- | --- | --- | --- | --- |
|  |  | **Right** | **Left** | **Right** | **Left** | **Right** | **Left** | **Right** | **Left** |
| LT | S4-S5 | 0 | 0 | 0 | 0 | 0 | 0 | 0 | 0 |
|  | **RLT + LLT** | **26** | **24** | **28** | **27** | **28** | **27** | **28** | **28** |
|  | **LT TOTAL** | **50** | | **55** | | **55** | | **56** | |
| **PP Prick** | S4-S5 | 0 | 0 | 0 | 0 | 0 | 0 | 0 | 0 |
|  | **RPP + LPP** | **26** | **25** | **26** | **23** | **27** | **27** | **26** | **24** |
|  | **PP TOTAL** | **51** | | **49** | | **54** | | **50** | |
|  | **DAP** | No | | No | | No | | No | |
|  | **VAC** | No | | No | | No | | No | |
|  |  |  |  |  |  |  |  |  | |
| **Upper extr.** | **C5 - Elbow flexors** | 5 | 5 | 5 | 5 | 5 | 5 | 5 | 5 |
|  | **C6 - Wrist extensors** | 5 | 5 | 5 | 5 | 5 | 5 | 5 | 5 |
|  | **C7 - Elbow extensors** | 5 | 5 | 5 | 5 | 5 | 5 | 5 | 5 |
|  | **C8 - Finger flexors** | 5 | 5 | 5 | 5 | 5 | 5 | 5 | 5 |
|  | **T1 - Finger abductors** | 5 | 5 | 5 | 5 | 5 | 5 | 5 | 5 |
|  |  |  |  |  |  |  |  |  |  |
| **Lower extr.** | **L2 - Hip flexors** | 0 | 0 | 0 | 0 | 0 | 0 | 0 | 0 |
|  | **L3 - Knee extensors** | 0 | 0 | 0 | 0 | 0 | 0 | 1 | 1 |
|  | **L4 - Ankle dorsiflexors** | 0 | 0 | 0 | 0 | 0 | 0 | 0 | 1 |
|  | **L5 - Long toe extensors** | 0 | 0 | 0 | 0 | 0 | 0 | 0 | 0 |
|  | **S1 - Ankle plantar flexors** | 0 | 0 | 0 | 0 | 0 | 0 | 0 | 0 |
|  |  |  |  |  |  |  |  |  | |
|  | **UER - UEL** | **25** | **25** | **25** | **25** | **25** | **25** | **25** | **25** |
|  | **UEMS TOTAL** | **50** | | **50** | | **50** | | **50** | |
|  | **LER - LEL** | **0** | **0** | **0** | **0** | **0** | **0** | **1** | **2** |
|  | **LEMS TOTAL** | **0** | | **0** | | **0** | | **3** | |
|  |  |  |  |  |  |  |  |  | |
| **Neuro. Levels** | **Sensory** | T5 | T5 | T5 | T4 | T5 | T5 | T5 | T5 |
|  | **Motor** | T5 | T5 | T5 | T4 | T5 | T5 | T5 | T5 |
|  | **Neurological Level of Injury** | T5 | | T4 | | T5 | | T5 | |
|  | **Complete or Incomplete** | C | | C | | C | | C | |
|  | **ASIA Impairment Scale** | A | | A | | A | | A | |
|  | **ZPP Sensory** | T7 | T6 | T9 | T9 | T9 | T8 | T9 | T9 |
|  | **ZPP Motor** | T5 | T5 | - | - | - | - | L3 | L4 |
|  | **Comments** |  | |  | |  | |  | |
|  |  |  | |  | |  | |  | |

**Table S7.** ISNCSCI assessment for participant P4 done at the onset of training (A1), at the end of the first block of 16 weeks of training (A2), and the end of the second block of 16 weeks of training (A3), and after an 8-week break (A4). LT: Light Touch; PP: Pin Prick; DAP: Deep anal pressure; VAC: Voluntary anal contraction; UER/UEL: Upper Extremity Right/Left; LER/LEL: Lower Extremity Right/Left; UEMS/LEMS: Upper/Lower Extremity Motor Score; ZPP: Zone of Partial Preservation.

|  |  | **A1** | | **A2** | | **A3** | | **A4** | |
| --- | --- | --- | --- | --- | --- | --- | --- | --- | --- |
|  |  | **Right** | **Left** | **Right** | **Left** | **Right** | **Left** | **Right** | **Left** |
| LT | S4-S5 | 0 | 0 | 0 | 0 | 0 | 0 | 0 | 0 |
|  | **RLT + LLT** | **32** | **32** | **35** | **35** | **36** | **35** | **34** | **34** |
|  | **LT TOTAL** | **64** | | **70** | | **71** | | **68** | |
| **PP Prick** | S4-S5 | 0 | 0 | 0 | 0 | 0 | 0 | 0 | 0 |
|  | **RPP + LPP** | **32** | **33** | **35** | **35** | **35** | **33** | **34** | **33** |
|  | **PP TOTAL** | **65** | | **70** | | **68** | | **67** | |
|  | **DAP** | No | | No | | No | | No | |
|  | **VAC** | No | | No | | No | | No | |
|  |  |  |  |  |  |  |  |  | |
| **Upper extr.** | **C5 - Elbow flexors** | 5 | 5 | 5 | 5 | 5 | 5 | 5 | 5 |
|  | **C6 - Wrist extensors** | 5 | 5 | 5 | 5 | 5 | 5 | 5 | 5 |
|  | **C7 - Elbow extensors** | 5 | 5 | 5 | 5 | 5 | 5 | 5 | 5 |
|  | **C8 - Finger flexors** | 5 | 5 | 5 | 5 | 5 | 5 | 5 | 5 |
|  | **T1 - Finger abductors** | 5 | 5 | 5 | 5 | 5 | 5 | 5 | 5 |
|  |  |  |  |  |  |  |  |  |  |
| **Lower extr.** | **L2 - Hip flexors** | 1 | 0 | 1 | 1 | 1 | 2 | 2 | 2 |
|  | **L3 - Knee extensors** | 0 | 0 | 1 | 1 | 1 | 1 | 1 | 1 |
|  | **L4 - Ankle dorsiflexors** | 0 | 0 | 0 | 0 | 0 | 0 | 0 | 0 |
|  | **L5 - Long toe extensors** | 0 | 0 | 0 | 0 | 0 | 0 | 0 | 1 |
|  | **S1 - Ankle plantar flexors** | 0 | 0 | 0 | 0 | 0 | 0 | 0 | 0 |
|  |  |  |  |  |  |  |  |  | |
|  | **UER - UEL** | **25** | **25** | **25** | **25** | **25** | **25** | **25** | **25** |
|  | **UEMS TOTAL** | **50** | | **50** | | **50** | | **50** | |
|  | **LER - LEL** | **1** | **0** | **2** | **2** | **2** | **3** | **3** | **4** |
|  | **LEMS TOTAL** | **1** | | **4** | | **5** | | **7** | |
|  |  |  |  |  |  |  |  |  | |
| **Neuro. Levels** | **Sensory** | T8 | T9 | T9 | T9 | T9 | T9 | T9 | T9 |
|  | **Motor** | T8 | T9 | T9 | T9 | T9 | T9 | T9 | T9 |
|  | **Neurological Level of Injury** | T8 | | T9 | | T9 | | T9 | |
|  | **Complete or Incomplete** | C | | C | | C | | C | |
|  | **ASIA Impairment Scale** | A | | A | | A | | A | |
|  | **ZPP Sensory** | T10 | T10 | T12 | T12 | L1 | T12 | T11 | T11 |
|  | **ZPP Motor** | L2 | T9 | L3 | L3 | L3 | L3 | L3 | L5 |
|  | **Comments** |  | |  | |  | |  | |

**Table S8.** ISNCSCI assessment for participant P5 done at the onset of training (A1), at the end of the first block of 16 weeks of training (A2), and the end of the second block of 16 weeks of training (A3), and after an 8-week break (A4). LT: Light Touch; PP: Pin Prick; DAP: Deep anal pressure; VAC: Voluntary anal contraction; UER/UEL: Upper Extremity Right/Left; LER/LEL: Lower Extremity Right/Left; UEMS/LEMS: Upper/Lower Extremity Motor Score; ZPP: Zone of Partial Preservation.

|  |  | **A1** | | **A2** | | **A3** | | **A4** | |
| --- | --- | --- | --- | --- | --- | --- | --- | --- | --- |
|  |  | **Right** | **Left** | **Right** | **Left** | **Right** | **Left** | **Right** | **Left** |
| LT | S4-S5 | 0 | 0 | 0 | 0 | 0 | 0 | 0 | 0 |
|  | **RLT + LLT** | **22** | **22** | **25** | **27** | **23** | **27** | **26** | **29** |
|  | **LT TOTAL** | **44** | | **52** | | **50** | | **55** | |
| **PP Prick** | S4-S5 | 0 | 0 | 0 | 0 | 0 | 0 | 0 | 0 |
|  | **RPP + LPP** | **22** | **22** | **25** | **25** | **23** | **27** | **29** | **30** |
|  | **PP TOTAL** | **44** | | **50** | | **50** | | **59** | |
|  | **DAP** | No | | No | | No | | No | |
|  | **VAC** | No | | No | | No | | No | |
|  |  |  |  |  |  |  |  |  | |
| **Upper extr.** | **C5 - Elbow flexors** | 5 | 5 | 5 | 5 | 5 | 5 | 5 | 5 |
|  | **C6 - Wrist extensors** | 5 | 5 | 5 | 5 | 5 | 5 | 5 | 5 |
|  | **C7 - Elbow extensors** | 5 | 5 | 5 | 5 | 5 | 5 | 5 | 5 |
|  | **C8 - Finger flexors** | 5 | 5 | 5 | 5 | 5 | 5 | 5 | 5 |
|  | **T1 - Finger abductors** | 5 | 5 | 5 | 5 | 5 | 5 | 5 | 5 |
|  |  |  |  |  |  |  |  |  |  |
| **Lower extr.** | **L2 - Hip flexors** | 0 | 0 | 0 | 0 | 0 | 0 | 0 | 0 |
|  | **L3 - Knee extensors** | 0 | 0 | 0 | 0 | 1 | 0 | 1 | 1 |
|  | **L4 - Ankle dorsiflexors** | 0 | 0 | 0 | 0 | 1 | 0 | 0 | 0 |
|  | **L5 - Long toe extensors** | 0 | 0 | 0 | 0 | 0 | 0 | 0 | 0 |
|  | **S1 - Ankle plantar flexors** | 0 | 0 | 0 | 0 | 0 | 0 | 0 | 0 |
|  |  |  |  |  |  |  |  |  | |
|  | **UER - UEL** | **25** | **25** | **25** | **25** | **25** | **25** | **25** | **25** |
|  | **UEMS TOTAL** | **50** | | **50** | | **50** | | **50** | |
|  | **LER - LEL** | **0** | **0** | **0** | **0** | **2** | **0** | **1** | **1** |
|  | **LEMS TOTAL** | **0** | | **0** | | **2** | | **2** | |
|  |  |  |  |  |  |  |  |  | |
| **Neuro. Levels** | **Sensory** | T3 | T3 | T3 | T3 | T3 | T3 | T4 | T4 |
|  | **Motor** | T3 | T3 | T3 | T3 | T3 | T3 | T4 | T4 |
|  | **Neurological Level of Injury** | T3 | | T3 | | T3 | | T4 | |
|  | **Complete or Incomplete** | C | | C | | C | | C | |
|  | **ASIA Impairment Scale** | A | | A | | A | | A | |
|  | **ZPP Sensory** | T5 | T5 | T8 | T10 | T7 | T10 | T11 | T12 |
|  | **ZPP Motor** | T3 | T3 | - | - | L4 | - | L3 | L3 |
|  | **Comments** |  | |  | |  | |  | |

**Table S9.** ISNCSCI assessment for participant P6 done at the onset of training (A1), at the end of the first block of 16 weeks of training (A2), and the end of the second block of 16 weeks of training (A3), and after an 8-week break (A4). LT: Light Touch; PP: Pin Prick; DAP: Deep anal pressure; VAC: Voluntary anal contraction; UER/UEL: Upper Extremity Right/Left; LER/LEL: Lower Extremity Right/Left; UEMS/LEMS: Upper/Lower Extremity Motor Score; ZPP: Zone of Partial Preservation.

|  |  | **A1** | | **A2** | | **A3** | | **A4** | |
| --- | --- | --- | --- | --- | --- | --- | --- | --- | --- |
|  |  | **Right** | **Left** | **Right** | **Left** | **Right** | **Left** | **Right** | **Left** |
| LT | S4-S5 | 0 | 0 | 0 | 0 | 0 | 0 | 0 | 0 |
|  | **RLT + LLT** | **39** | **36** | **38** | **37** | **41** | **39** | **39** | **39** |
|  | **LT TOTAL** | **75** | | **75** | | **80** | | **78** | |
| **PP Prick** | S4-S5 | 0 | 0 | 0 | 0 | 0 | 0 | 0 | 0 |
|  | **RPP + LPP** | **39** | **36** | **38** | **37** | **40** | **37** | **40** | **39** |
|  | **PP TOTAL** | **75** | | **75** | | **77** | | **79** | |
|  | **DAP** | No | | No | | Yes | | Yes | |
|  | **VAC** | No | | No | | No | | Yes | |
|  |  |  |  |  |  |  |  |  | |
| **Upper extr.** | **C5 - Elbow flexors** | 5 | 5 | 5 | 5 | 5 | 5 | 5 | 5 |
|  | **C6 - Wrist extensors** | 5 | 5 | 5 | 5 | 5 | 5 | 5 | 5 |
|  | **C7 - Elbow extensors** | 5 | 5 | 5 | 5 | 5 | 5 | 5 | 5 |
|  | **C8 - Finger flexors** | 5 | 5 | 5 | 5 | 5 | 5 | 5 | 5 |
|  | **T1 - Finger abductors** | 5 | 5 | 5 | 5 | 5 | 5 | 5 | 5 |
|  |  |  |  |  |  |  |  |  |  |
| **Lower extr.** | **L2 - Hip flexors** | 0 | 0 | 1 | 0 | 1 | 1 | 2 | 2 |
|  | **L3 - Knee extensors** | 0 | 0 | 0 | 1 | 1 | 1 | 1 | 1 |
|  | **L4 - Ankle dorsiflexors** | 0 | 0 | 0 | 0 | 0 | 0 | 0 | 0 |
|  | **L5 - Long toe extensors** | 0 | 0 | 0 | 0 | 0 | 0 | 0 | 0 |
|  | **S1 - Ankle plantar flexors** | 0 | 0 | 0 | 0 | 0 | 0 | 0 | 0 |
|  |  |  |  |  |  |  |  |  | |
|  | **UER - UEL** | **25** | **25** | **25** | **25** | **25** | **25** | **25** | **25** |
|  | **UEMS TOTAL** | **50** | | **50** | | **50** | | **50** | |
|  | **LER - LEL** | **0** | **0** | **1** | **1** | **2** | **2** | **3** | **3** |
|  | **LEMS TOTAL** | **0** | | **2** | | **4** | | **6** | |
|  |  |  |  |  |  |  |  |  | |
| **Neuro. Levels** | **Sensory** | T11 | T10 | T10 | T10 | T11 | T11 | T11 | T11 |
|  | **Motor** | T11 | T10 | T10 | T10 | T11 | T11 | T11 | T11 |
|  | **Neurological Level of Injury** | T10 | | T10 | | T11 | | T11 | |
|  | **Complete or Incomplete** | C | | C | | I | | I | |
|  | **ASIA Impairment Scale** | A | | A | | C | | C | |
|  | **ZPP Sensory** | L2 | T12 | L2 | L1 | S3 | S3 | S3 | L2 |
|  | **ZPP Motor** | T11 | T10 | L2 | L3 | L3 | L3 | L3 | L3 |
|  | **Comments** |  | |  | | Deep anal pressure present, and motor function present more than three segments (L4) below motor level (T11) | | Deep anal pressure present and voluntary anal contraction | |

**Table S10.** ISNCSCI assessment for participant P7 done at the onset of training (A1), at the end of the first block of 16 weeks of training (A2), and the end of the second block of 16 weeks of training (A3), and after an 8-week break (A4). LT: Light Touch; PP: Pin Prick; DAP: Deep anal pressure; VAC: Voluntary anal contraction; UER/UEL: Upper Extremity Right/Left; LER/LEL: Lower Extremity Right/Left; UEMS/LEMS: Upper/Lower Extremity Motor Score; ZPP: Zone of Partial Preservation.

|  |  | **A1** | | **A2** | | **A3** | | **A4** | |
| --- | --- | --- | --- | --- | --- | --- | --- | --- | --- |
|  |  | **Right** | **Left** | **Right** | **Left** | **Right** | **Left** | **Right** | **Left** |
| LT | S4-S5 | 0 | 0 | 0 | 0 | 0 | 0 | 0 | 0 |
|  | **RLT + LLT** | **31** | **30** | **34** | **35** | **37** | **38** | **40** | **42** |
|  | **LT TOTAL** | **61** | | **69** | | **75** | | **82** | |
| **PP Prick** | S4-S5 | 0 | 0 | 0 | 0 | 0 | 0 | 0 | 0 |
|  | **RPP + LPP** | **31** | **30** | **33** | **34** | **34** | **33** | **35** | **35** |
|  | **PP TOTAL** | **61** | | **67** | | **67** | | **70** | |
|  | **DAP** | No | | No | | No | | Yes | |
|  | **VAC** | No | | No | | No | | Yes | |
|  |  |  |  |  |  |  |  |  | |
| **Upper extr.** | **C5 - Elbow flexors** | 5 | 5 | 5 | 5 | 5 | 5 | 5 | 5 |
|  | **C6 - Wrist extensors** | 5 | 5 | 5 | 5 | 5 | 5 | 5 | 5 |
|  | **C7 - Elbow extensors** | 5 | 5 | 5 | 5 | 5 | 5 | 5 | 5 |
|  | **C8 - Finger flexors** | 5 | 5 | 5 | 5 | 5 | 5 | 5 | 5 |
|  | **T1 - Finger abductors** | 5 | 5 | 5 | 5 | 5 | 5 | 5 | 5 |
|  |  |  |  |  |  |  |  |  |  |
| **Lower extr.** | **L2 - Hip flexors** | 0 | 0 | 0 | 0 | 0 | 1 | 0 | 1 |
|  | **L3 - Knee extensors** | 0 | 0 | 0 | 0 | 1 | 0 | 1 | 1 |
|  | **L4 - Ankle dorsiflexors** | 0 | 0 | 0 | 0 | 0 | 0 | 0 | 0 |
|  | **L5 - Long toe extensors** | 0 | 0 | 0 | 0 | 0 | 0 | 0 | 0 |
|  | **S1 - Ankle plantar flexors** | 0 | 0 | 0 | 0 | 0 | 0 | 0 | 0 |
|  |  |  |  |  |  |  |  |  | |
|  | **UER - UEL** | **25** | **25** | **25** | **25** | **25** | **25** | **25** | **25** |
|  | **UEMS TOTAL** | **50** | | **50** | | **50** | | **50** | |
|  | **LER - LEL** | **0** | **0** | **0** | **0** | **1** | **1** | **1** | **2** |
|  | **LEMS TOTAL** | **0** | | **0** | | **2** | | **3** | |
|  |  |  |  |  |  |  |  |  | |
| **Neuro. Levels** | **Sensory** | T8 | T7 | T9 | T9 | T8 | T8 | T9 | T9 |
|  | **Motor** | T8 | T7 | T9 | T9 | T8 | T8 | T9 | T9 |
|  | **Neurological Level of Injury** | T7 | | T9 | | T8 | | T9 | |
|  | **Complete or Incomplete** | C | | C | | C | | I | |
|  | **ASIA Impairment Scale** | A | | A | | A | | C | |
|  | **ZPP Sensory** | T9 | T9 | T11 | T12 | L2 | S3 | S3 | S3 |
|  | **ZPP Motor** | T8 | T7 | - | - | L3 | - | L3 | L3 |
|  | **Comments** |  | |  | |  | | Deep anal pressure present, voluntary anal contraction and motor function present more than three levels (L4) below motor level (T9) | |

**Table S11.** ISNCSCI assessment for participant P8 done at the onset of training (A1), at the end of the first block of 16 weeks of training (A2), and the end of the second block of 16 weeks of training (A3), and after an 8-week break (A4). LT: Light Touch; PP: Pin Prick; DAP: Deep anal pressure; VAC: Voluntary anal contraction; UER/UEL: Upper Extremity Right/Left; LER/LEL: Lower Extremity Right/Left; UEMS/LEMS: Upper/Lower Extremity Motor Score; ZPP: Zone of Partial Preservation.

|  |  | **A1** | | **A2** | | **A3** | | **A4** | |
| --- | --- | --- | --- | --- | --- | --- | --- | --- | --- |
|  |  | **Right** | **Left** | **Right** | **Left** | **Right** | **Left** | **Right** | **Left** |
| LT | S4-S5 | 0 | 0 | 0 | 0 | 0 | 0 | 0 | 0 |
|  | **RLT + LLT** | **34** | **34** | **35** | **35** | **35** | **36** | **34** | **36** |
|  | **LT TOTAL** | **68** | | **70** | | **71** | | **70** | |
| **PP Prick** | S4-S5 | 0 | 0 | 0 | 0 | 0 | 0 | 0 | 0 |
|  | **RPP + LPP** | **35** | **34** | **34** | **37** | **34** | **34** | **34** | **35** |
|  | **PP TOTAL** | **69** | | **71** | | **68** | | **69** | |
|  | **DAP** | No | | No | | No | | No | |
|  | **VAC** | No | | No | | No | | No | |
|  |  |  |  |  |  |  |  |  | |
| **Upper extr.** | **C5 - Elbow flexors** | 5 | 5 | 5 | 5 | 5 | 5 | 5 | 5 |
|  | **C6 - Wrist extensors** | 5 | 5 | 5 | 5 | 5 | 5 | 5 | 5 |
|  | **C7 - Elbow extensors** | 5 | 5 | 5 | 5 | 5 | 5 | 5 | 5 |
|  | **C8 - Finger flexors** | 5 | 5 | 5 | 5 | 5 | 5 | 5 | 5 |
|  | **T1 - Finger abductors** | 5 | 5 | 5 | 5 | 5 | 5 | 5 | 5 |
|  |  |  |  |  |  |  |  |  |  |
| **Lower extr.** | **L2 - Hip flexors** | 0 | 0 | 0 | 1 | 1 | 1 | 0 | 1 |
|  | **L3 - Knee extensors** | 0 | 0 | 1 | 1 | 0 | 1 | 0 | 1 |
|  | **L4 - Ankle dorsiflexors** | 0 | 0 | 1 | 1 | 0 | 1 | 0 | 1 |
|  | **L5 - Long toe extensors** | 0 | 0 | 0 | 0 | 0 | 0 | 0 | 0 |
|  | **S1 - Ankle plantar flexors** | 0 | 0 | 0 | 0 | 0 | 0 | 0 | 0 |
|  |  |  |  |  |  |  |  |  | |
|  | **UER - UEL** | **25** | **25** | **25** | **25** | **25** | **25** | **25** | **25** |
|  | **UEMS TOTAL** | **50** | | **50** | | **50** | | **50** | |
|  | **LER - LEL** | **0** | **0** | **2** | **3** | **1** | **3** | **0** | **3** |
|  | **LEMS TOTAL** | **0** | | **5** | | **4** | | **3** | |
|  |  |  |  |  |  |  |  |  | |
| **Neuro. Levels** | **Sensory** | T9 | T9 | T9 | T9 | T9 | T10 | T9 | T10 |
|  | **Motor** | T9 | T9 | T9 | T9 | T9 | T10 | T9 | T10 |
|  | **Neurological Level of Injury** | T9 | | T9 | | T9 | | T9 | |
|  | **Complete or Incomplete** | C | | C | | C | | C | |
|  | **ASIA Impairment Scale** | A | | A | | A | | A | |
|  | **ZPP Sensory** | T12 | T11 | T12 | L2 | T12 | T12 | T11 | S3 |
|  | **ZPP Motor** | T9 | T9 | L4 | L4 | L2 | L4 | - | L4 |
|  | **Comments** |  | |  | |  | |  | |

**Supplementary Figures**

**
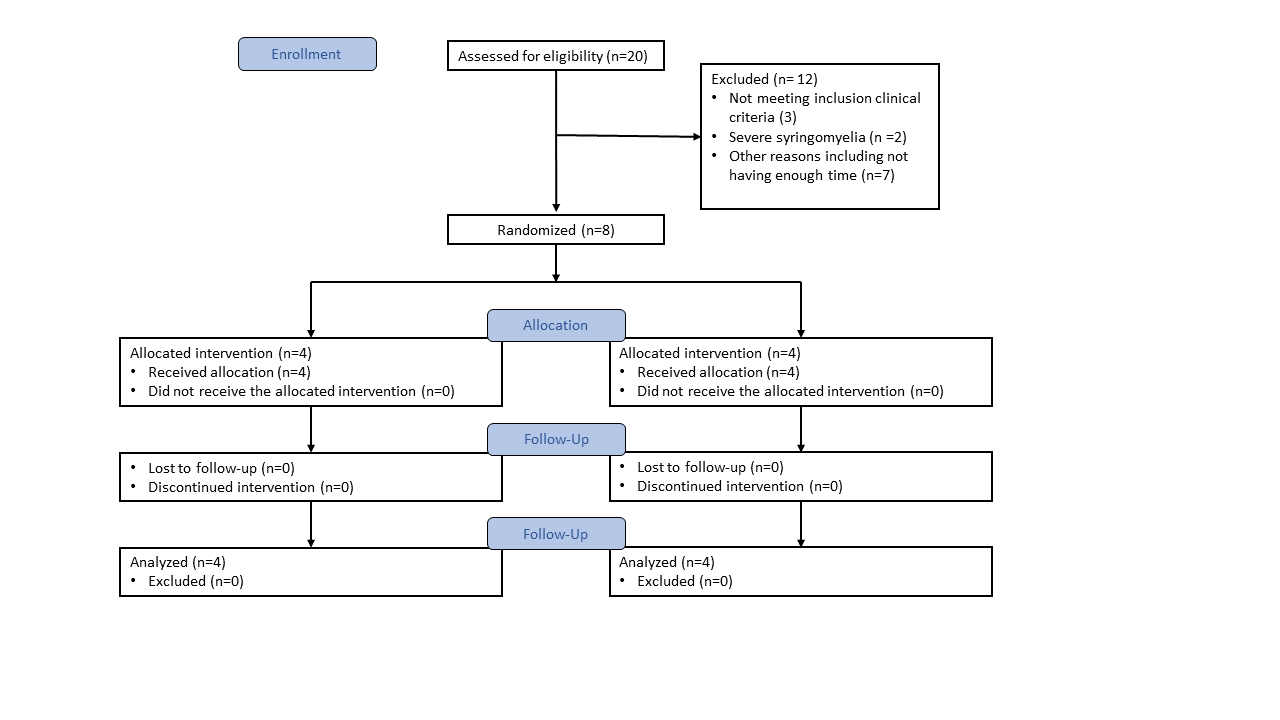
**

**Figure S1.** Participant flow diagram.


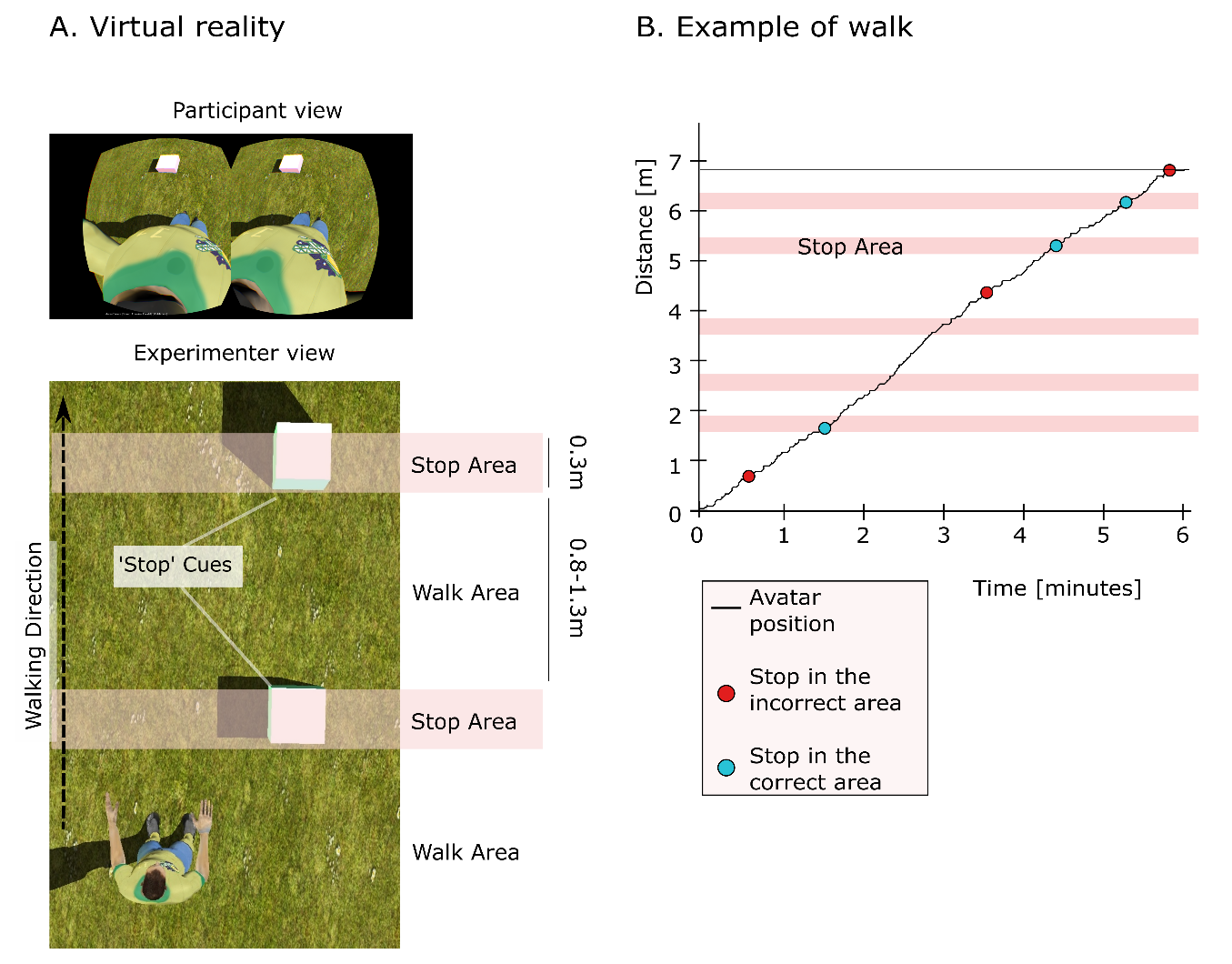


**Fig. S2 BMI Performance calculation.** (**A**) (top) The avatar was projected in a first-person perspective in the head-mounted display. (bottom) The Experimenter’s view of the avatar and the stop cues randomly placed in the environment (spaced by 80 and 130cm).

(**B**) Example walk performed during a full 6-minute session: pink areas are regions where the participant should stop the avatar at least one time. Blue dots are stop events that occurred inside the ‘Stop area’ and red dots describe cases where the subject stopped the avatar in a walk area. The performance score, termed, corrected walked distance (CWD) is defined by

$$CWD=D-(mC)$$

Where D is the distance walked during the 6 minutes block, m the number of stop areas where the participant did not manage to stop, and C is the size of the stop cues in meter. On the example shown here, D = 6.8m, the participant missed two stop areas (starting from the bottom, respectively the second and the third stop area), and C is fixed to 0.3m (the cue size). Therefore, the CWD for this run was:

$$6.8m-2*0.3m=6.2m$$

**
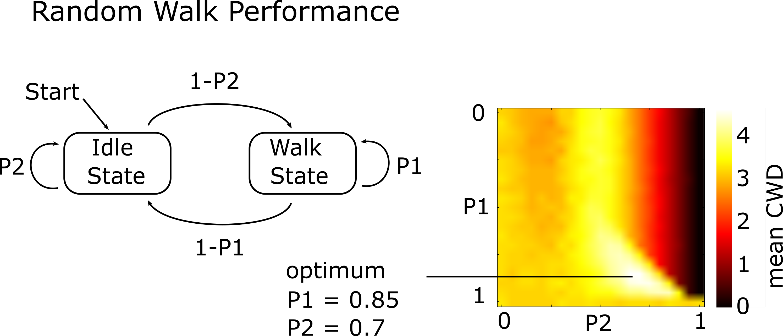
**

**Figure S3. Systematic search for the parameters for the random walk.** The chance level was estimated using a two-state Markov chain, which is defined as a group of finite state random processes where the probability of changing states depends only on the actual one. A random walk was then defined by the probability of staying in the walk state (P1) and the probability of staying in the stop state (P2). The probabilities of changing from walk to stop is given by 1-P1 and the probability of changing from stop to walk by 1-P2. To find the pair that gave the maximum random walk performance, we tested all combinations of P1 and P2 ranging from 0 to 1 (with an indentation of 0.05) and calculated the correct walked distance for 100 randomly generated walks based on the probability pair. The mean CWD was calculated for each walk based on P1, and P2. The maximum mean random performance was given by P1 = 0.85 and P2 = 0.70. The corresponding CWD was 4.40m$\pm$0.63 m (mean$\pm$STD). The maximum possible CWD was equal to 7.45m. It was found by playing a regular session but deliberately controlling the avatar to stop only one time at the right place.

**Supplementary** **movies**

**Movie S1.** Example of BMI trial. (Top) Patient’s view of the avatar seen from first person perspective. (bottom) Experimenter view of the classifier. Threshold for this experiment was set to 1. Right or left step animations were triggered when the classifier was respectively above Threshold or below -Threshold. When the classifier value was in the [-Threshold +Threshold] range,

To give online feedback on the classifier sign, we added a small shaking animation on the left leg when the classifier value was in the [-Threshold 0] range (respectively the on right leg when the classifier values was in the [0 Threshold] range.

**Movie S2.** Example of motor examination for P6 at A2, A3 and A4. Participant was asked to perform a right leg abduction.
