# Supplementary material for: Training with noninvasive brain–machine interface, tactile feedback, and locomotion to enhance neurological recovery in individuals with complete paraplegia: a randomized pilot study
Source: Sci Rep. 2022 Nov 29;12:20545. doi: 10.1038/s41598-022-24864-5 (PMC9709065; doi:10.1038/s41598-022-24864-5)
Supplement: Supplementary file 2 — Supplementary Information 2. [file 41598_2022_24864_MOESM2_ESM.pdf]

A

		MEDIAN						TIBIAL					C7 ROOT		
		N20	P25	P14	N13	ERB	A	P40	N50	P40-N50	N32	N21	N20	P25	A
**P1**	LEFT	21,20	22,70	13,30	13,50	11,40	1,00	0,00	0,00	0,00	0,00	20,70	24,90	27,90	-2,90
	RIGHT	20,80	23,20	13,30	13,40	11,10	0,90	0,00	0,00	0,00	0,00	21,30	24,30	27,60	-3,30
**P2**	LEFT	17,70	20,20	13,60	11,80	9,60	2,20	0,00	0,00	0,00	0,00	0,00	21,30	25,60	-4,30
	RIGHT	19,10	22,20	13,80	13,20	9,90	1,10	0,00	0,00	0,00	0,00	0,00	22,80	25,70	-2,80
**P3**	LEFT	20,10	22,20	13,70	14,00	11,00	0,40	0,00	0,00	0,00	0,00	0,00	23,80	28,30	-4,50
	RIGHT	20,00	24,90	12,70	13,50	11,30	1,10	0,00	0,00	0,00	0,00	0,00	26,00	30,00	-4,00
**P4**	LEFT	20,50	25,20	13,70	14,90	11,20	2,20	0,00	0,00	0,00	0,00	0,00	24,00	29,20	-5,20
	RIGHT	20,80	25,60	15,20	14,70	11,60	2,60	0,00	0,00	0,00	0,00	0,00	25,90	30,80	-4,90
**P5**	LEFT	19,20	21,90	14,10	13,30	10,90	2,60	0,00	0,00	0,00	0,00	23,30	23,20	27,20	-4,00
	RIGHT	19,30	22,00	13,20	11,70	10,80	4,90	0,00	0,00	0,00	0,00	21,00	21,50	25,70	-4,30
**P6**	LEFT	18,20	20,70	12,60	11,80	9,10	2,00	0,00	0,00	0,00	0,00	0,00	19,50	22,80	-3,30
	RIGHT	17,80	20,50	12,50	12,10	9,30	3,70	0,00	0,00	0,00	0,00	0,00	20,40	24,00	-3,60
**P7**	LEFT	19,50	22,20	13,80	12,90	10,30	3,80	0,00	0,00	0,00	0,00	0,00	22,80	26,50	-3,70
	RIGHT	18,90	21,70	14,40	13,90	10,30	3,20	0,00	0,00	0,00	0,00	0,00	22,40	27,20	-4,80
**P8**	LEFT	20,00	23,30	12,80	13,50	10,40	0,40	0,00	0,00	0,00	0,00	0,00	22,10	25,30	-3,20
	RIGHT	20,00	22,00	13,50	14,10	10,20	0,40	0,00	0,00	0,00	0,00	0,00	22,80	26,50	-3,70
