# Supplementary material for: Training with noninvasive brain–machine interface, tactile feedback, and locomotion to enhance neurological recovery in individuals with complete paraplegia: a randomized pilot study
Source: Sci Rep. 2022 Nov 29;12:20545. doi: 10.1038/s41598-022-24864-5 (PMC9709065; doi:10.1038/s41598-022-24864-5)
Supplement: Supplementary file 3 — Supplementary Information 3. [file 41598_2022_24864_MOESM3_ESM.pdf]

			T1			T2			T3			T4			T5			T6			T7			T8			T9			T10			T11			T12			L1		L2		L3		L4		L5	
			P40	N50	A	P40	N50	A	P40	N50	A	P40	N50	A	P40	N50	A	P40	N50	A	P40	N50	A	P40	N50	A	P40	N50	A	P40	N50	A	P40	N50	A	P40	N50	A										
**P1**	**LOC**	LEFT													34,20	38,80	0,30				0,00	0,00	0,00	0,00	0,00	0,00				0,00	0,00	0,00														0,00	0,00
		RIGHT													26,80	30,00	0,30				0,00	0,00	0,00	0,00	0,00	0,00				0,00	0,00	0,00														0,00	0,00
**P2**	**LBMI**	LEFT																						30,20	35,30	0,20	0,00	0,00	0,00	0,00	0,00	0,00	0,00	0,00	0,00											0,00	0,00
		RIGHT																						29,60	34,70	0,50	0,00	0,00	0,00	0,00	0,00	0,00	0,00	0,00	0,00											0,00	0,00
**P3**	**LOC**	LEFT										21,80	25,50	0,10				40,50	45,10	0,20																0,00	0,00	0,00									0,00	0,00
		RIGHT										21,70	24,30	0,10				42,70	49,60	0,10																0,00	0,00	0,00									0,00	0,00
**P4**	**LBMI**	LEFT																			27,80	36,50	1,10	30,10	36,50	0,00				0,00	0,00	0,00	0,00	0,00	0,00											0,00	0,00
		RIGHT																			28,30	38,70	1,10	29,60	33,10	0,20				0,00	0,00	0,00	0,00	0,00	0,00											0,00	0,00
**P5**	**LOC**	LEFT							26,50	29,80	0,20	30,90	35,50	0,20	31,00	33,50	0,20	0,00	0,00	0,00																											0,00	0,00
		RIGHT							24,50	29,30	0,20	0,00	0,00	0,00	0,00	0,00	0,00	0,00	0,00	0,00																											0,00	0,00
**P6**	**LBMI**	LEFT																									25,20	32,30	0,30	29,60	42,10	0,30				0,00	0,00	0,00			0,00	0,00	0,00	0,00			0,00	0,00
		RIGHT																									26,80	30,80	0,10	30,70	40,00	0,30				0,00	0,00	0,00			0,00	0,00	0,00	0,00			0,00	0,00
**P7**	**LBMI**	LEFT																33,90	37,60	0,20	33,90	42,90	0,30				0,00	0,00	0,00	0,00	0,00	0,00															0,00	0,00
		RIGHT																34,40	38,70	0,30	38,40	33,10	0,10				0,00	0,00	0,00	0,00	0,00	0,00															0,00	0,00
**P8**	**LOC**	LEFT																42,10	46,40	0,10	X						46,10	56,80	0,20	0,00	0,00	0,00	0,00	0,00	0,00											0,00	0,00
		RIGHT																46,40	51,70	0,10	X						37,30	41,90	0,10	0,00	0,00	0,00	0,00	0,00	0,00											0,00	0,00
